# A comprehensive CFD lifecycle dataset for marine vessel hydrodynamics

**DOI:** 10.1038/s41597-026-07490-y

**Published:** 2026-06-13

**Authors:** Song Wang, Chen Wang, Jianchun Wang, Xin Yu, Zhe Xu, Shengguo Li, Linjie Zhang, Yuchao Guo, Jun Ding

**Affiliations:** 1 Energy and Internet Research Institute, Tsinghua University, Beijing, China; 2 National Engineering Research Center for Big Data Software, Tsinghua University, Beijing, China; 3 Ship and Marine Technology Innovation Center, China Ship Scientific Research Center, Beijing, China; 4 College of Computer Science and Technology, National University of Defense Technology, Beijing, China; 5 School of Mathematics and Computational Science, Xiangtan University, Beijing, China; 6 National Key Laboratory of Strength and Structural Integrity, Aircraft Strength Research Institute of China, Beijing, China; 7 National Elite Institute of Engineering, Northwestern Polytechnical University, Beijing, China

**Keywords:** Scientific data, Computational science, Computer science

## Abstract

Recent advances in artificial intelligence offer considerable promise for enhancing industrial computational simulation workflows, yet practical adoption is limited by a shortage of high-quality, real-world datasets. Most existing CFD-related datasets focus on a single phase of the simulation lifecycle, typically post-processed flow snapshots, and lack the upstream information needed to train models that can exploit the full pipeline. In this work, we present three such datasets, collected from the China Ship Scientific Research Center during operational industrial workflows. The data cover three marine vessels-two tanker ships and a submarine-whose low-speed, high-block-coefficient hull forms produce complex, separated stern flows. Uniquely, and in contrast to conventional repositories, our dataset spans the complete CFD lifecycle: pre-processing (experimental documentation and geometries), solving (the linear systems **A*x*** = ***b*** resulting from discretisation), and post-processing (numerical solutions and computational meshes). By assembling these interdependent stages into a single, coherent resource, the dataset is designed to support the development of robust AI models that can learn across phases, while also facilitating research in solver performance evaluation, data management, and flow visualization. Beyond AI, the dataset serves a wider range of research topics, such as benchmark-driven solver evaluation, data-management studies, and flow-field visualization, enhancing its overall utility and impact.

## Background & Summary

The design of modern aircraft, vessels, and ground vehicles relies heavily on computational simulations for performance optimisation, with CFD (C omputational F luid D ynamics) serving as a critical application^1–4^. As digital simulation techniques become more advanced, there is an increasing demand for more precise computational outcomes using meshes of finer granularity, particularly for intricate simulation challenges. Consequently, the execution of modern computational simulations can be computationally intensive, often necessitating days or even weeks to yield a high-quality result. In response to the substantial time investment required, the research and engineering communities are actively pursuing alternative approaches to enhance the effectiveness and efficiency of computational simulation processes^3–5^.

The development of AI technology in the field of computational simulation tasks brings new light to the efficient and effective solutions towards complex simulation tasks. Representative use cases include parameter selection^6^, neural operator^7,8^, mesh generation & optimization^9^, PINNs (Physics-Informed Neural Networks)^10,11^, etc. Among these use cases, PINNs has drawn great research interest recently. Unlike traditional solutions that directly solve a large linear equation system, PINNs approaches tend to *predict* the outcome of such equations via neural networks, which is much more efficient. With proper training, PINNs can produce very precise outcomes. Such features draw great interest in the recent development of the field of computational physics^12–15^.

Nevertheless, to effectively train and apply AI technology to computational simulation, one requires a large amount of training data. However, due to the intricacy of the principle of the computational simulation experiment, such an experiment involves many factors, including governing equations, boundary conditions, discretized meshes, solvers, computed results, etc. All these factors could potentially influence the training process of an AI model. Nevertheless, most of the publicly available datasets fail to cover all the aspects, leading to unsatisfactory utilization of AI technology on real-life computational simulation applications.

Most of the existing datasets are derived from relatively naive simulation tasks with simple geometrical features and regular governing equations (such as poission and laplacian)^16^. The numerical features of these datasets do not align with real simulation tasks encountered in actual engineering. Public matrix datasets like SuiteSparse^17^ merely include the matrix input of the computational task, without the theoretical background and groundtruth results. By far the most SOTA (State-Of-The-Art) dataset is the Well^18^ dataset, which manages to enclose the initial matrix and the solutions generated by the iterative methods. However, the theoretical background of the task remains missing.

The missing factors of existing datasets could block the potential development of AI technology in the field of computational simulation. For example, with the knowledge of the governing equations and mesh characteristics, researchers could categorize datasets into classes, and train different PINNs models designed for solving simulation problems for ships, submarines, aircraft, etc. Potentially available techniques like this require as much information about the simulation task as possible.

In this paper, we present a dataset comprising three CFD simulation cases. All data presented in this work is generated using MarineFlow (https://www.taihulab.com.cn/einfo/210.html), a CFD software developed by CSSRC (China Ship Scientific Research Center) (http://www.cssrc.com.cn/). Our dataset focuses on three CFD experiments on three ship types, two tanker ships and a submarine. The scales of the CFD models are summarized in Table 1. Each case is supplied with the complete lifecycle information generated during the computational experiment: pre-processing setups, solving-phase matrices, and post-processing results.

**Table 1:** Summary of the datasets.

Ship Model	Speed (*m*/*s*)	Mesh Scale
**Kvlcc2**	1.050	351, 000
		3, 709, 000
**Suboff**	3.051	528, 000
		3, 258, 000
**JBC**	1.179	615, 000
		3, 843, 000

Apart from its rich content, our dataset is grounded in CFD cases with practical industrial relevance. In ship hydrodynamic performance analysis and design, both full-scale and model-scale experiments are conducted at high Reynolds numbers, where the flow is predominantly turbulent. The multiscale, strongly sheared, and highly nonlinear nature of turbulence makes direct numerical simulation prohibitively expensive for routine engineering. As a result, turbulence-resolving or turbulence-modelled CFD has become the standard approach for reliably predicting ship resistance, propulsion characteristics, and complex flow-field structures at an acceptable computational cost. To faithfully capture this industrial need, the marine vessels in this study—two tanker ships and one submarine—are designed to operate in a low-speed, high-block hydrodynamic regime (Froude number broadly in the range 0.1–0.4, Reynolds number on the order of 10^6^–10^7^), representative of full-scale merchant and naval craft. The complexity of such flow scenarios poses a considerable challenge for AI-driven solvers and surrogate models, positioning our dataset as a demanding and realistic benchmark for the development of robust AI-based simulation tools.

We anticipate that researchers will use this rich, multi-phase dataset to improve the training of AI models for computational simulation, leading to faster and more accurate predictions. By bridging the gap between academic AI research and real-world industrial workflows, this data has the potential to accelerate the adoption of AI-driven tools in engineering practice.

## Methods

This section describes the CFD methodology and data generation process underlying the datasets. All simulations follow established best practices for ship hydrodynamics, specifically the procedures recommended by the ITTC (International Towing Tank Conference) for resistance and self-propulsion tests (https://www.ittc.info/media/8169/75-03-03-01.pdf). All CFD simulations were performed with the incompressible RANS (Reynolds-Averaged Navier-Stokes) solver using a steady-state formulation. The convective terms in the momentum equations were discretised with a second-order upwind scheme, while the diffusion terms used a second-order central-difference scheme. Pressure-velocity coupling was handled by the SIMPLE algorithm. Turbulence closure was achieved with the SST *k*–*ω* model^19^, employing standard model constants and an automatic wall function for near-wall treatment. Convergence was judged by a six-order-of-magnitude drop in the normalized momentum and pressure residuals, together with the stabilization of the total ship resistance. This choice of standard solver and turbulence model ensures that the experimental setups are reproducible and reflect widely accepted industrial CFD practice.

For the sake of clarity, the following conventions are used throughout the paper to distinguish between matrices, vectors, and scalars: Matrices are represented by bold, uppercase letters (e.g., **A**), vectors by bold, lowercase letters (e.g., ***u***), and scalars by regular lowercase letters (e.g., *ρ*).

### Principle of CFD

**Fig. 1:**
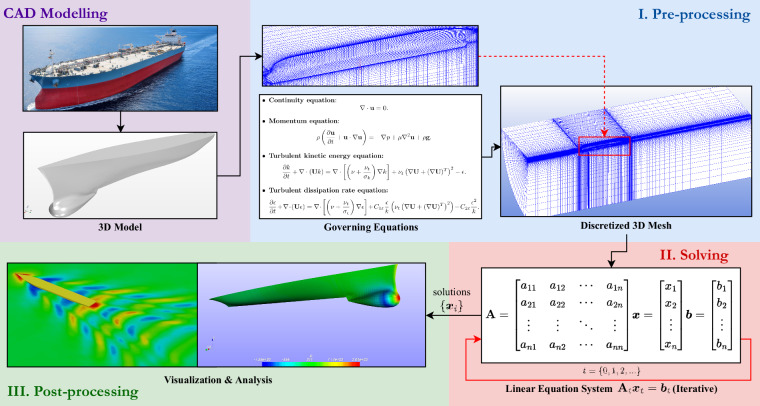
The lifecycle of a CFD task.

This section briefly introduces the workflow of a CFD simulation. Figure 1 illustrates a representative lifecycle of a CFD experiment. While the underlying principles are well known to domain specialists, we provide a simple yet complete example to supply the necessary background for readers from other fields.

Because the goal of a marine CFD simulation is to reproduce real-life ship performance, the first step is to create a digital model of the vessel—in particular, the part below the waterline where the hull interacts with the surrounding fluid. The construction of this three-dimensional geometry belongs to the domain of CAD (Computer Aided Design), which is beyond the scope of this paper; however, the resulting 3D model constitutes an essential input for the CFD experiment.

#### Pre-processing

The pre-processing phase of CFD involves constructing a **mesh** from the 3-D ship model. This mesh, denoted ${\mathscr{M}}$, consists of a large number of discrete cells that cover the vessel and the surrounding water body, as illustrated in Fig. 1. To simulate the flow, the governing partial differential equations (such as the Reynolds-averaged Navier-Stokes equations) are then applied to the mesh through **discretization**. Discretization replaces the continuous equations with a system of algebraic equations that relate the flow quantities in neighbouring cells; the specific form of these relationships is determined by the chosen numerical schemes. The assembly of these cell-wise equations yields the large sparse linear systems that constitute the solving-phase data, a process that will be explained in the following subsection.

#### Solving

The discretization produces a sparse linear system for each set of physical variables, written compactly as

1 $${\bf{A}}{\boldsymbol{x}}={\boldsymbol{b}}.$$

The solving phase is dedicated to solving these systems. In this work, the pressure-velocity coupling in the incompressible Navier-Stokes equations is handled by the SIMPLE (Semi-Implicit Method for Pressure-Linked Equations) algorithm^20^.

**Fig. 2:**
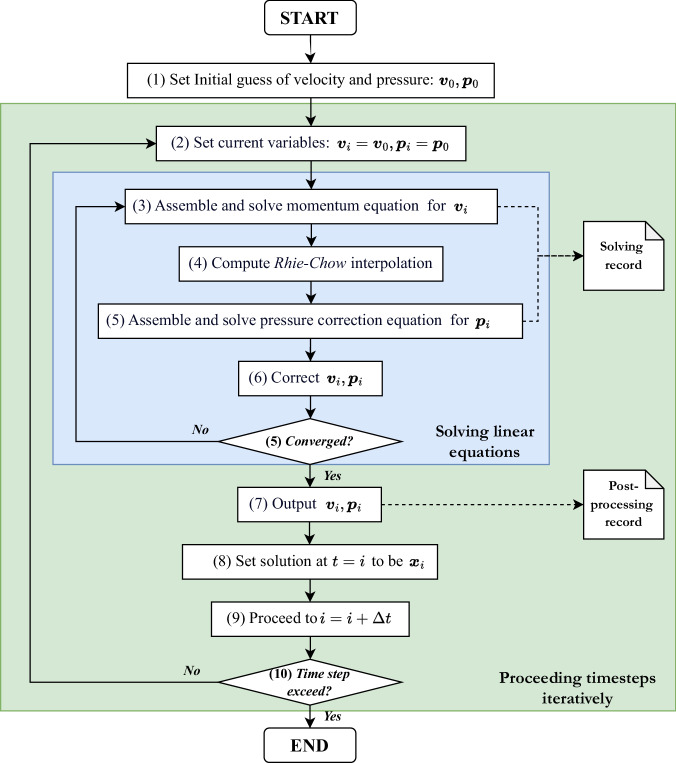
The flowchart of the SIMPLE algorithm.

Figure 2 illustrates the iterative structure of the SIMPLE algorithm. Two nested loops are highlighted: the inner loop (blue) represents the assembly and solution of the linear systems **A*x*** = ***b*** for each physical variable, yielding the solving-phase data; the outer loop (green) corresponds to the time-step advancement. Each converged outer iteration produces a flow-field snapshot, which collectively constitutes the post-processing data.

From the structure of the SIMPLE algorithm, the diversity of the linear systems arises from two orthogonal sources: the physical variables being solved for, and the time discretization of the transient simulation. We denote the set of physical variable types by ${\mathscr{V}}$ and the set of discrete time steps by ${\mathscr{T}}$.

- **Domain**${\mathcal{V}}$: A CFD simulation tracks multiple fields, e.g. velocity components {***u***, ***v***, ***w***}, pressure ***p***, and turbulence quantities {***k***, ***e***}. Each field is governed by a distinct transport equation, which, upon discretisation, yields a separate linear system. In the following, we use a superscript $v\in {\mathscr{V}}$ to distinguish these systems: **A**^*v*^***x***^*v*^ = ***b***^*v*^.
- **Domain**${\mathscr{T}}$: Transient CFD simulations split a continuous physical process (e.g., a ship sailing for 2 000 ms) into a sequence of discrete time steps, e.g. ${\mathscr{T}}=\{100,200,300,\ldots ,2000\}$ ms. Because the flow at one time step influences the next through wave propagation and advection, the discretised equations, and hence the matrix **A**^*v*^ and right-hand side ***b***^*v*^, change with each time step. We therefore extend the notation to ${{\bf{A}}}_{t}^{v}{{\boldsymbol{x}}}_{t}^{v}={{\boldsymbol{b}}}_{t}^{v}$ for a given time $t\in {\mathscr{T}}$ and variable $v\in {\mathscr{V}}$.

The Cartesian product of the two domains yields a total of $| {\mathscr{V}}| \times | {\mathscr{T}}|$ distinct linear systems,

2 $$S=\{{{\bf{A}}}_{t}^{v}{{\boldsymbol{x}}}_{t}^{v}={{\boldsymbol{b}}}_{t}^{v}\,| \,t\in {\mathscr{T}},\,v\in {\mathscr{V}}\},$$

which together constitute the solving-phase dataset.

#### Post-processing

Solving each linear system in the solving-phase dataset yields numerical approximations of the physical fields for every cell at each simulated time step. The collection of these solution vectors,

3 $${\mathcal{X}}=\{{{\boldsymbol{x}}}_{t}^{v}| t\in {\mathscr{T}},\,v\in {\mathscr{V}}\},$$

constitutes the core of the post-processing phase. From ${\mathcal{X}}$ one can compute engineering quantities of interest: for instance, the velocity field can be analyzed to trace the formation and dissipation of vortices, and the pressure distribution can be integrated over the hull surface to determine the total hydrodynamic resistance. These analytical tasks all belong to the realm of post-processing and are entirely dependent on the solution set ${\mathcal{X}}$. To aid interpretation, researchers often overlay the solution data on the computational mesh, as illustrated in Fig. 1. For this reason, the geometrical mesh topology is typically included together with ${\mathcal{X}}$ in the post-processing dataset.

### From PDE to Linear Equation Systems

This section outlines how the governing partial differential equations are discretised into a system of linear equations expressed in matrix form, **A*x*** = ***b***. Understanding this transformation is essential because it directly links the three data phases: the structure, sparsity pattern, and entries of the coefficient matrix **A** are determined by the pre-processing setup (geometry, mesh, and physical parameters); the process of assembling and solving the linear systems constitutes the solving-phase data; and the resulting solution vectors ***x*** form the core of the post-processing data.

The fundamental purpose of CFD is to simulate real-life physical processes. To achieve this, CFD software must adhere to the physical laws governing these processes. These laws are encapsulated in equations known as governing equations, which are typically expressed as PDEs (Partial Differential Equation). These equations are central to CFD simulations and are essential for capturing the complex interactions within fluid flow. A prime example of such governing equations is the N-S equations, which are utilized to describe the behavior of fluids. The N-S equations for incompressible fluid are expressed as follows:

$$\begin{array}{c}\nabla \cdot {\boldsymbol{u}}=0,\\ \rho \frac{{\rm{D}}{\boldsymbol{u}}}{{\rm{D}}t}=-\nabla \cdot p+\nabla \cdot {\boldsymbol{\tau }}+\rho {\bf{F}}.\end{array}$$

These two equations correspond to the conservation of mass and momentum, respectively. For simplicity, we focus on the conservation of mass, i.e.

4 $$\nabla \cdot {\boldsymbol{u}}=0.$$

Symbol  ∇ represents computing the partial derivative on each dimension. In a three-dimensional space, we have

5 $$\nabla =\frac{\partial }{\partial x}+\frac{\partial }{\partial y}+\frac{\partial }{\partial z}.$$

***u*** represents velocity, which is a three-dimensional vector

6 $${\boldsymbol{u}}=[u,v,w],$$

where *u*, *v*, and *w* corresponds to the *x*, *y*, and *z* components of ***u***, respectively.

Combining equations (4), (5) and (6), we have

7 $$\frac{\partial u}{\partial x}+\frac{\partial v}{\partial y}+\frac{\partial w}{\partial z}=0.$$

An intuitive explanation to equation (7) is that, for any given position within the fluid, if the fluid is flowing out in one direction (such as *x*), the fluid flows in the other directions (*y* and *z*) will adjust to compensate for this loss, to ensure that the overall quantity of fluid is conserved, i.e. the conservation of mass.

According to the principle of partial differentiation, by discretizing the overall spatial domain into a sufficiently fine grid of small cells, it is possible to approximate partial derivatives using a cell-wise computational approach. This process is known as *discretization* and is a fundamental technique in CFD.

**Fig. 3:**
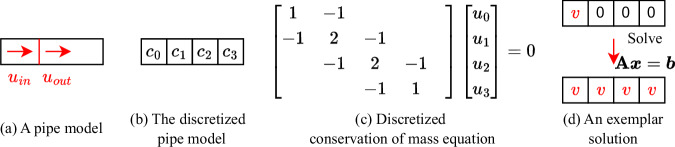
A simple example of the principle of CFD discretization.

For instance, if we discretize the pipe into four equally-spaced cells (as depicted in Fig. 3 subfigure (b)), the PDE governing fluid flow can be approximated by a system of linear equations for each cell. Specifically, considering *c*_0_, we have

$$\frac{\partial {u}_{0}}{\partial x}=\frac{{u}_{1}-{u}_{0}}{{c}_{1}-{c}_{0}}=0\Rightarrow {u}_{1}-{u}_{0}.$$

Similarly, for *c*_1_, we can derive

8 $$\frac{{u}_{1}-{u}_{0}}{{c}_{1}-{c}_{0}}-\frac{{u}_{2}-{u}_{1}}{{c}_{2}-{c}_{1}}=0\Rightarrow 2{u}_{1}-{u}_{0}-{u}_{2}=0,$$

etc. Combining these linear equations form a linear equation system **A*u*** = 0, demonstrated in subfigure (c). In other words, **A*u*** = 0 is the *discretized form* of the PDE  ∇ ***u*** = 0. We typically express the linear equation system in a more generalized form **A*x*** = ***b***, where ***b*** is known as the boundary condition.

The discretization process described above represents a single step in the broader context of CFD simulations. In practical applications, the initial conditions are not static but evolve over time. This evolution is critical for modeling dynamic scenarios such as a ship accelerating in water. As the ship accelerates, the velocity of the fluid cells adjacent to its body will increase over time. To capture this temporal evolution, a CFD simulation task discretizes time into time steps. Each time step represents a small interval during which the fluid flow is assumed to remain constant. At each time step, the discretized model is updated to reflect the changes in the initial conditions due to the evolving velocity field.

### Data Types

Each of the three discinctive phases of a CFD task generates. These data are varied in forms, yet intinsically related in theory. Specifically, the pre-processing phase takes initial setups and the ship model as input, and generates discretized linear equations in the form of matrices as output. The solving phases solves these equations and generates solution vectors. The post-processing phase combines the solution vectors with the ship mesh for post-processing analytical tasks. In this section, we will explain the structures of data at each phase.

#### Pre-processing data

Pre-processing encompasses the setup of the CFD problem, including the choice of governing equations, the geometry of the 3-D ship model, and the specification of physical operating conditions (water density, speed, etc.). A typical set of physical parameters is

$$\begin{array}{l}\rho =998.8\,{\rm{kg}}/{{\rm{m}}}^{3};\\ \mu =0.001116\,{\rm{Pa}}\cdot {\rm{s}};\\ u=1.179\,{\rm{m}}/{\rm{s}};\\ v=0.0\,{\rm{m}}/{\rm{s}};\\ w=0.0\,{\rm{m}}/{\rm{s}},\end{array}$$

where *ρ* and *μ* denote the water density and dynamic viscosity, respectively, and *u*, *v*, *w* are the three components of the ship’s velocity. These parameters describe the physical configuration of the CFD scenario; further details are contained in the pre-processing data files.

This information is mostly descriptive rather than structured. We therefore store it as a document, with the 3D model saved in a separate, standard .cas file. Each CFD case in the dataset contains exactly one document and one 3D model file.

**Fig. 4:**
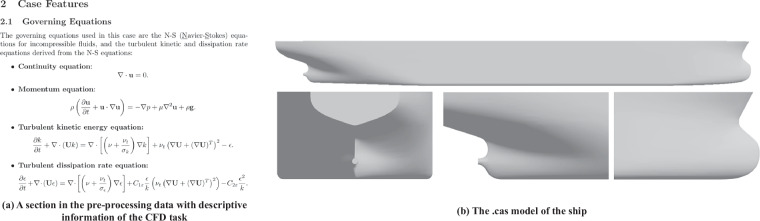
The exemplar content of a pre-processing data record.

Figure 4 illustrates an example pre-processing record, which consists of two components:

- A document describing the background of the CFD experiment-the governing equations, the physical parameter values, the list of variables to be solved, and similar metadata. Subfigure (a) reproduces the portion of the document that specifies the governing equations employed in the CFD task.
- A .cas file that contains the 3-D ship geometry model, as is displayed in subfigure (b).

Pre-processing data are essential for extending the utility of our dataset. In PINNs, for example, CFD problems with similar configurations-such as comparable ship types, physical parameters, and governing equations-can potentially be solved by a single trained model^21^. In linear algebra, knowledge of the problem origin provides important clues for selecting appropriate solver settings and preconditioners^22^. Existing matrix collections^17,18^ largely omit this contextual information, which limits the potential to develop and optimize algorithms that could exploit it.

#### Solving data

The solving phase comprises the sequence of sparse linear systems

9 $${{\bf{A}}}_{t}^{v}{{\boldsymbol{x}}}_{t}^{v}={{\boldsymbol{b}}}_{t}^{v},$$

where $v\in {\mathcal{V}}$ indexes the physical variable (velocity component, pressure, etc.) and $t\in {\mathscr{T}}$ the discrete time step. For each simulation case, our dataset provides both the coefficient matrices ${{\bf{A}}}_{t}^{v}$ and the corresponding right-hand side (RHS) vectors ${{\boldsymbol{b}}}_{t}^{v}$. Owing to the iterative time-marching scheme and the need to solve for multiple physical fields, a single CFD run generates exactly $| {\mathscr{V}}| \times | {\mathscr{T}}|$ such systems. The dimension of each matrix and vector is of order $| {\mathscr{M}}|$, i.e., the total number of cells in the computational mesh.

**Fig. 5:**
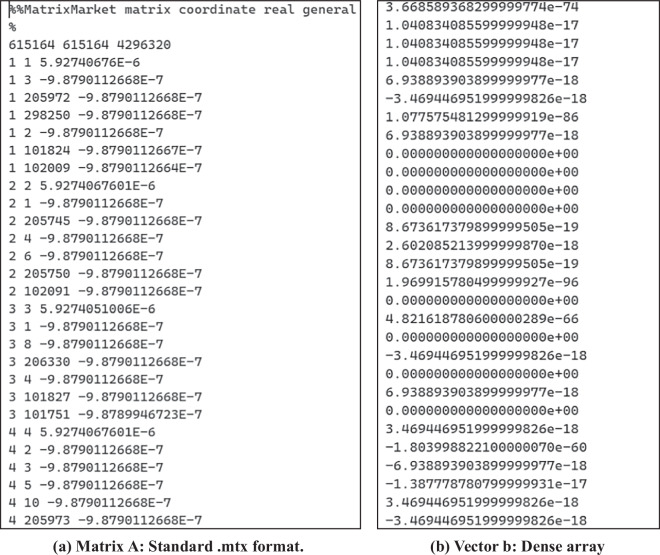
Example content of a solving data record.

Figure 5 displays an example solving-phase matrix and its RHS vector, stored in the standard Matrix Market (.mtx) format. These records can be read with widely available libraries; alternatively, users may write custom parsers because the matrices are internally stored in the coordinate (COO) format^23^. In a COO file, the first two columns hold the row and column indices, respectively, while the third column contains the value of the non-zero entry. The RHS vector is stored as a single-column dense array.

Critically, the mesh topology ${\mathscr{M}}$ is equation-agnostic: it is determined only by the geometry of the computational domain, not by the specific form of the governing PDEs. Mesh resolution directly governs simulation fidelity: finer meshes improve the approximation of the PDEs but incur higher computational cost. Consequently, the size of ${\mathscr{M}}$ (denoted $| {\mathscr{M}}|$) is constrained by the available hardware. On typical engineering workstations, $| {\mathscr{M}}|$ in the range 10^5^-10^7^ offers a practical compromise between accuracy and resource consumption. High-performance computing facilities can accommodate $| {\mathscr{M}}|$ up to 10^9^, enabling large-scale simulations of geometrically complex configurations.

From a graph-theoretic viewpoint, each matrix ${{\bf{A}}}_{t}^{v}$ can be interpreted as a weighted adjacency matrix of the discretised mesh: a non-zero entry ${({{\bf{A}}}_{t}^{v})}_{ij}$ encodes the discrete interaction between cells *i* and *j*. This perspective reveals several structural properties that distinguish CFD matrices from generic sparse matrices:

- **Sparsity**. As illustrated in Fig. 3, each cell communicates only with a small, local set of neighbours. Thus the adjacency matrix is inherently sparse, typically containing no more than 10 non-zeros per row regardless of $| {\mathscr{M}}|$.
- **Balanced non-zero distribution**. High-quality CFD meshes are constructed to be as uniform as possible across the domain, leading to a roughly constant number of adjacent cells for every interior node. Consequently, the number of non-zeros per row is nearly homogeneous. Local mesh refinement near complex geometric features (e.g., the bow and stern of the ship) introduces moderate row-wise variations, yet the overall non-zero count per row remains well balanced.
- **Constant sparsity pattern**. Because the computational mesh in our dataset is static throughout the simulation, the sparsity pattern-the set of (*i*, *j*) coordinate pairs-is identical for all matrices $\{{{\bf{A}}}_{t}^{v}\}$ from a single case. Only the numerical values of the entries change with time and physical variable. More advanced CFD approaches, such as adaptive mesh refinement, would produce a time-dependent sparsity structure; this is beyond the scope of the present dataset.
- **Diagonal dominance**. The rows of the matrices are generally diagonally dominant,
  10 $$| {({{\bf{A}}}_{t}^{v})}_{ii}| \,\ge \,\sum _{j\ne i}| {({{\bf{A}}}_{t}^{v})}_{ij}| ,$$
  a property inherited from the discretization of elliptic operators and the use of upwind-type stabilization. In many rows the inequality is strict, guaranteeing favourable iterative solver convergence.
- **Diversity**. A distinctive strength of our dataset compared to existing sparse-matrix collections is its diversity along two axes. With $| {\mathscr{V}}| =6$ physical variables and $| {\mathscr{T}}| =10$ time steps, each CFD experiment contributes 60 related but distinct matrices. This structured variety allows researchers to study how matrix properties evolve with physical quantity and simulation time, and to develop or benchmark algorithms that exploit such controlled heterogeneity.

#### Post-processing data

**Fig. 6:**
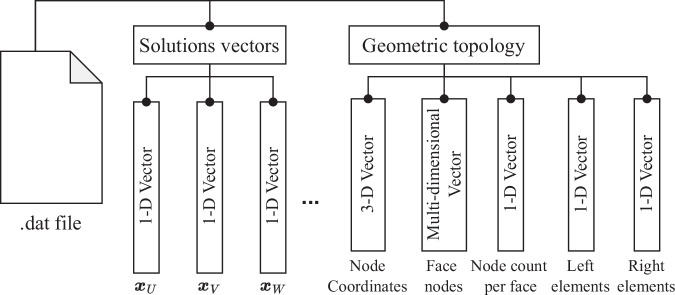
Components of a post-processing data file.

The post-processing phase is built upon the solution sets $\{{{\boldsymbol{x}}}_{t}^{v}\}$ obtained by solving all linear systems ${{\bf{A}}}_{t}^{v}{{\boldsymbol{x}}}_{t}^{v}={{\boldsymbol{b}}}_{t}^{v}$ from the solving phase. However, most post-processing tasks-such as computing global forces or visualizing flow features-require the solutions to be mapped back onto the computational mesh. Therefore, the post-processing data also include the geometric topology of the discretized ship model, as illustrated in Fig. 6. Both the solution fields and the mesh connectivity are stored in vector form within the data files.

The files follow the standard .dat format, which is widely used by commercial and open-source post-processing tools (e.g., Tecplot, ParaView, MarineFlow). Each .dat file contains the solution of all $| {\mathscr{V}}|$ physical variables on a single time step $t\in {\mathscr{T}}$. Consequently, the size of one post-processing file scales as $O(| {\mathscr{M}}| \times | {\mathscr{V}}| )$, and the dataset includes $| {\mathscr{M}}|$ such files.

**Fig. 7:**
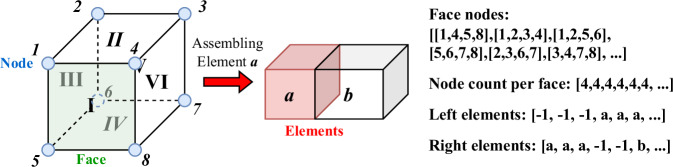
Assembly of the discretized mesh topology from the post-processing data.

While the interpretation of solution vectors is straightforward, reconstructing a three-dimensional mesh from the topology vectors stored in a .dat file is less obvious. We therefore provide a detailed example of the assembly procedure. Figure 7 illustrates a minimal mesh composed of two elements, *a* and *b*, and traces the construction of element *a*. The process begins with the *Node coordinates* array (see Fig. 6), which defines isolated points in space. In our example, element *a* has eight nodes, labelled with Arabic numerals {1, 2, …, 8}.

The next step constructs the faces by specifying which nodes form each face. For clarity we use Roman numerals {*I*, *II*, …, *VI*} for the faces. Face *I*, for instance, is formed by nodes {1, 4, 5, 8}, producing the first entry of the **FN** (Face Nodes) array. Repeating this for all faces yields the complete FN array.

The **NCPF** (Node Count Per Face) array records the number of nodes belonging to each face. In this regular mesh every face has four nodes, so NCPF is uniform; in more complex meshes NCPF values may vary. The NCPF array serves as an **offset** that enables parsing of the FN array, because in practice the FN data are stored as a single one-zimensional array rather than as a list of sub-arrays.

Once the faces are defined, the **LE** (Left Element) and **RE** (Right Element) arrays specify which element lies on each side of a face. For faces *I* through *V*, which lie on the boundary, one element is *a* and the other is set to  −1, indicating no adjacent element. Face *VI* is internal, shared by element *a* and element *b*; its LE entry is *a* and its RE entry is *b*. This pairing establishes that elements *a* and *b* are neighbours across face *VI*.

By combining the node coordinates, FN, NCPF, LE and RE arrays, one can reconstruct the full three-dimensional mesh together with its complete connectivity information.

Given the centrality of post-processing data in interpreting simulation outcomes, we detail its hierarchical structure and parsing methodology. Our open-source tool, publicly available on GitHub (https://github.com/ft1573734/Dat_Data_Decoder), formalizes the data into three domains: time, variables, and meshes. This tripartite structure enables systematic querying, visualization, and validation of simulation results against experimental or operational data.

## Data Records

The CFD lifecycle dataset^24^ is released on the Science Data Bank (https://www.scidb.cn/) under CC-BY 4.0 licence, and can be accessed via the following doi link: 10.57760/sciencedb.23682. The dataset comprises six compressed files, each representing a batch of the lifecycle data of the CFD experiment on a ship type.

**Fig. 8:**
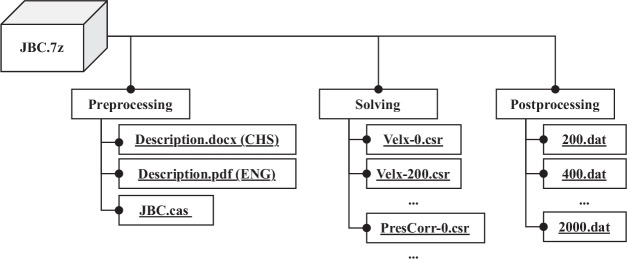
The hierarchy of an uploaded dataset.

Due to the large scale of the original dataset, each batch of experimental results is compressed into a single zip file. The architecture of the compressed file is illustrated in Fig. 8. Files in the dataset are underlined. We briefly introduce the content under each folder.

- **Pre-processing**. The Pre-processing folder contains the setup information of the simulation experiment, with a descriptive document and a .cas file representing the geometrical information of the data. The .cas file can be read using AutoCAD (https://www.autodesk.com/).
- **Solving**. The Solving folder contains the matrices and RHS at each timestep. The original data is encapsulated in a .csr file, which is organized using the logic of CSR (Compressed Sparse Row), a common sparse matrix format. We provide an open-source script to decode the .csr file into a regular *matrix market*^25^ file format (.mtx), since .mtx is more commonly used in matrix processing, and there have been plenty of existing libraries, in Python (https://docs.scipy.org/doc/scipy/reference/generated/scipy.io.mmread.html), C/C++ (https://git.sr.ht/~cwpearson/matrix-market), etc., that use .mtx file as data exchange interfaces. Our script is released on GitHub (https://github.com/ft1573734/Matrix_Converter).
- **Post-processing**. The Post-processing folder contains the post-processing data files in .dat format, which is compatible with post-processing software such as Tecplot (https://tecplot.com/). We implemented a script for decoding the .dat files into arrays of different variables. Our script is released on Github (https://github.com/ft1573734/Dat_Data_Decoder).

## Technical Validation

We validate the dataset from two complementary perspectives: mathematical consistency and physical accuracy.

First, we examine the **mathematical properties** of the solving-phase data (the matrices {**A**}) to verify that the structural features described in the *Data Types* section-sparsity, balanced non-zero distribution, constant pattern, diagonal dominance, and diversity-are consistently exhibited across all cases and time steps.

Second, we assess the **physical fidelity** of the post-processing results (the solution fields {***x***}) by comparing them against both a state-of-the-art commercial CFD solver (StarCCM+) and high-quality physical towing-tank measurements. This dual comparison demonstrates that the numerical solutions produced by our MarineFlow pipeline are accurate and industrially relevant.

### Mathematical property validation

Our mathematical analysis prioritizes evaluating matrix properties for two key reasons. First, matrices are computationally central to the simulation workflow: solving the linear system ${{\bf{A}}}_{t}^{v}{{\boldsymbol{x}}}_{t}^{v}={{\boldsymbol{b}}}_{t}^{v}$ for each variable $v\in {\mathscr{V}}$ and timestep $t\in {\mathscr{T}}$ involves repeated matrix assembly, factorization, and iteration. Second, established datasets, e.g., SuiteSparse^17^, universally emphasize matrix features to quantify solver performance and stability. Beyond benchmarking, these properties hold practical value: They inform algorithm parameter selection (e.g., preconditioner choice, iteration tolerances) and guide architecture design for PINNs, where matrix spectra and sparsity patterns directly influence training efficiency and solution fidelity.

Tables 2, 3, and 4 demonstrate the mathematical features we evaluated for the matrices corresponding to all physical variables, at the first iteration of the three ship types on the smaller meshes. The larger meshes hold similar features which we omit in the paper. The features of the larger matrices can be computed using the code we release on Github (https://github.com/ft1573734/Matrix_Features), or using publicly available tools such as Octave (https://octave.org/) or Matlab (https://www.mathworks.com/).

**Table 2:** Mathematical Features of JBC Matrices.

Features	JBC-Velx	JBC-Vely	JBC-Velz	JBC-PresCorr	JBC-TurbK	JBC-TurbE
Row Count	615164	615164	615164	615164	615164	615164
Column Count	615164	615164	615164	615164	615164	615164
NNZ Count	4296320	4296320	4296320	4296320	4296320	4177269
Sparsity	1.13*e*^−5^	1.13*e*^−5^	1.13*e*^−5^	1.13*e*^−5^	1.13*e*^−5^	1.10*e*^−5^
Diagonally-dominant row count	614479	614480	614479	365055	614527	614527
Ratio of diagonally-dominant rows	99.9%	99.9%	99.9%	59.3%	99.9%	100%
Minimum nnz row count	4	4	4	4	4	1
Maximum nnz row count	16	16	16	16	16	16

**Table 3:** Mathematical Features of Kvlcc2 Matrices.

Features	Kvlcc2-Velx	Kvlcc2-Vely	Kvlcc2-Velz	Kvlcc2-PresCorr	Kvlcc2-TurbK	Kvlcc2-TurbE
Row Count	351864	351864	351864	351864	351864	351864
Column Count	351864	351864	351864	351864	351864	351864
NNZ Count	2426070	2426070	2426070	2426070	2426070	2404015
Sparsity	1.95*e*^−5^	1.95*e*^−5^	1.95*e*^−5^	1.95*e*^−5^	1.95*e*^−5^	1.94*e*^−5^
Diagonally-dominant row count	351864	351864	351864	180255	351864	351864
Ratio of diagonally-dominant rows	100%	100%	100%	51.2%	100%	100%
Minimum nnz row count	4	4	4	4	4	1
Maximum nnz row count	7	7	7	7	7	7

**Table 4:** Mathematical Features of Suboff Matrices.

Features	Suboff-Velx	Suboff-Vely	Suboff-Velz	Suboff-PresCorr	Suboff-TurbK	Suboff-TurbE
Row Count	528407	528407	528407	528407	528407	528407
Column Count	528407	528407	528407	528407	528407	528407
NNZ Count	3689407	3689407	3689407	3689407	3689407	3581559
Sparsity	1.32*e*^−5^	1.32*e*^−5^	1.32*e*^−5^	1.32*e*^−5^	1.32*e*^−5^	1.28*e*^−5^
Diagonally-dominant row count	527239	527225	527227	307089	527555	528407
Ratio of diagonally-dominant rows	99.7%	99.7%	99.7%	58.1%	99.8%	100%
Minimum nnz row count	4	4	4	4	4	1
Maximum nnz row count	17	17	17	17	7	17

In our dataset, all the experiments computes six unique physical variables, constituting domain ${\mathcal{V}}$:

- **Velx:** The *x* component of velocity.
- **Vely:** The *y* component of velocity.
- **Velz:** The *z* component of velocity.
- **PresCorr:** Pressure correction factor.
- **TurbK:** Turbulent kinetic energy.
- **TurbE:** Turbulence dissipation rate.

The definition of the mathematical features we extracted in the tables are as follows:

- **Row count**. The number of rows of the matrix.
- **Column count**. The number of columns of the matrix.
- **NNZ count**. The number of NNZs of the matrix.
- **Sparsity**. The sparsity of the matrix.
- **Diagonally-dominant row count**. The number of diagonally dominant, rows of the matrix, i.e., ∣**A**_*ii*_∣ > ∑∣**A**_*ij*_∣ where *i* ≠ *j*.
- **Ratio of diagonally-dominant rows**. The ratio of diagonally dominant rows.
- **Minimum nnz row count**. The minimum of NNZ count per row.
- **Maximum nnz row count**. The maximum of NNZ count per row.

The values reported in the tables confirm that the structural features identified in Section *Data Types* apply to nearly all matrices in the dataset. We discuss each feature in turn.

**Sparsity:** All matrices, regardless of ship type or physical variable, are extremely sparse. The sparsity ratio (number of non-zeros divided by total entries) is on the order of 10^−5^ in every case, a characteristic shared by every CFD-derived matrix in our collection.

**Balanced non-zero distribution:** This property is evident from the *Minimum nnz row count* and *Maximum nnz row count* entries. The gap between the minimum and maximum non-zero counts per row is very small (~10), especially when compared with the total number of rows (>10^5^). The non-zeros are therefore distributed nearly uniformly across rows.

**Shape consistency:** For a given ship type, the total non-zero count is almost identical across the matrices belonging to different physical variables; only the matrix corresponding to *TurbE* (turbulent kinetic energy) shows a slight deviation. This demonstrates that the sparsity pattern is essentially the same for all physical variables.

**Diagonal dominance:** The fraction of diagonally dominant rows shows that all matrices, apart from those representing the pressure correction (*PresCorr*), have more than 99.9% of their rows diagonally dominant. Even the *PresCorr* matrices exceed 50%, so we can safely conclude that the majority of matrices in the dataset exhibit strong diagonal dominance.

**Diversity:** Diversity is not captured by any single table entry, but rather by the overall statistical composition of the dataset. Our data stem from three marine vessel types, each with two mesh resolutions, yielding six distinct CFD cases. Each case records $| {\mathscr{V}}| =6$ physical variables over $| {\mathscr{T}}| =10$ time steps, resulting in a total of 360 complete linear systems **A*x*** = ***b*** provided in standard formats. All systems originate from real industrial CFD simulations, and the structured variation across geometry, variable type, and simulation time constitutes the diversity claimed.

### Physical fidelity validation

We evaluate the physical accuracy of the simulation results by comparing the MarineFlow predictions against two independent references: (i) a state-of-the-art commercial CFD solver (StarCCM+), and (ii) high-fidelity physical towing-tank experiments. The experimental measurements serve as the ground truth in all comparisons; therefore, numerical results that are in closer agreement with the experimental data are considered more reliable.

#### Velocity contour analysis

To validate the numerical accuracy of the CFD simulation results, we adopt the JBC (\underline{J}apan \underline{B}ulk \underline{C}arrier), an 180,000 DWT capesize bulk carrier, as the test case. This choice is motivated by the following considerations. The JBC is a typical low-speed, full-form vessel with a large block coefficient, representing one of the three principal merchant ship types. The full bow and midship lines, combined with a sharply tapered stern, readily induce flow separation and complex vortex systems, resulting in a highly complicated stern flow field. Owing to its low speed and full hull form, the generated wave lengths are relatively short, and the bow waves are prone to breaking, exhibiting strong nonlinearity. These characteristics pose significant challenges for numerical simulation and represent key difficulties in the independent development of ship hydrodynamic CFD software.

**Fig. 9:**
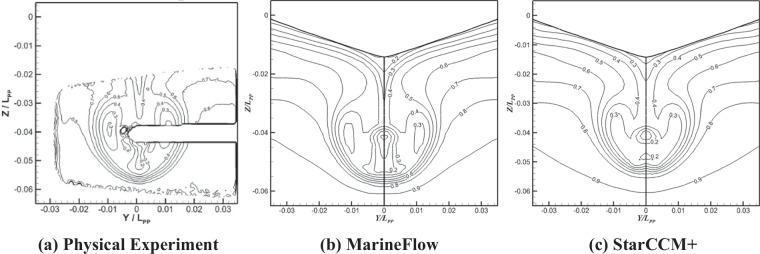
Streamwise velocity contours at a cross-section of the JBC ship model, obtained by physical experiment, MarineFlow, and StarCCM+.

Figure 9 presents the streamwise velocity contours at the cross-section *x*/*L*_PP_ = 0.9843 (*L*_PP_: length between perpendiculars) of the JBC model. Subfigure (a) shows the velocity contour at the stern S4 section in the without-propeller and without-duct condition, originally published by the NMRI (National Maritime Research Institute) of Japan in 2015^26^. This contour was obtained using a stereoscopic particle image velocimetry system, which derives the flow field from tracer particle images. Subfigures (b) and (c) display the corresponding computational results obtained with MarineFlow and StarCCM+, respectively.

Both numerical methods capture the overall structure of the stern flow field. In the high-velocity region (*u* ≥ 0.5), the predicted contour patterns are generally consistent with the experimental results. In the bilge-vortex region, MarineFlow reproduces the vortex structure more accurately, yielding the shape and location of the low-speed contour (*u* = 0.3) in closer agreement with the experiment and reasonably capturing the local vortex induced by the bilge. By contrast, StarCCM+ exhibits noticeable numerical diffusion in this region and fails to clearly resolve the vortex structure. In the region above *u* = 0.4 near the centerline, both methods underestimate the velocity to varying degrees. The velocity field in this zone is closely associated with stern flow separation and reattachment, processes that are inherently unsteady and difficult for steady RANS models to capture, revealing a fundamental limitation in predicting complex wake flows. For the low-speed region beneath the hull (*u* = 0.2 ~ 0.3), the extent of the contour predicted by MarineFlow is reasonably close to the experiment, albeit with a slight overall overestimation, whereas StarCCM+ fails to adequately capture this low-velocity structure, indicating an insufficient prediction of flow separation.

#### Resistance analysis

This part of the validation evaluates the hydrodynamic resistance experienced by the ship under the prescribed operating conditions. The MarineFlow predictions are compared against both the results of the commercial solver StarCCM+ and the physical towing-tank measurements conducted at CSSRC, which serve as the ground truth. Table 5 presents the comparison. The column headings are defined as follows:

- **Model**: the vessel model under consideration.
- **Speed**: the towing speed (or inflow velocity) of the vessel.
- **Solver**: the computational software used.
- **Mesh Scale**: the scale or approximate cell count of the computational mesh.
- **Computed Resistance**: the total hydrodynamic resistance predicted by the simulation; differences between solvers and mesh configurations arise from discretisation errors and mesh-dependent effects.
- **Real Resistance**: the total resistance measured in the physical towing-tank experiment (ground truth).
- **Error**: the relative deviation of the computed resistance from the experimental value.

**Table 5:** Experimental Result Validation.

Model	Speed (*m*/*s*)	Solver	Mesh Scale	Resistence		
				Computed Resistance (*N*)	Real Resistance (*N*)	Error
**JBC**	1.179	MarineFlow	615, 000	34.470	35.110	− 1.8%
			3, 843, 000	34.845		− 0.8%
		StarCCM+	615, 000	32.779		− 6.6%
			3, 843, 000	34.017		− 3.1%
**Kvlcc2**	1.050	MarineFlow	351, 000	18.162	18.487	− 1.8%
			3, 709, 000	18.260		− 1.2%
		StarCCM+	351, 000	18.747		1.4%
			3, 709, 000	18.265		− 1.2%
**Suboff**	3.051	MarineFlow	528, 000	108.659	102.300	6.2%
			3, 258, 000	99.456		− 2.8%
		StarCCM+	528, 000	94.760		− 7.4%
			3, 258, 000	94.719		− 7.4%

From Table 5, it can be seen that the MarineFlow predictions consistently agree well with the experimental measurements. For the JBC and KVLCC2 models, the relative error is within ±1.8%; for the SUBOFF model, the error remains within ±6.2%. These results confirm the accuracy of the MarineFlow solver and, by extension, the quality of the post-processing data provided in the dataset.

Taken together, the velocity-contour comparison and the resistance analysis show that the MarineFlow simulations are in closer agreement with the physical experiments than those obtained with the commercial baseline solver StarCCM+. This outcome validates the numerical accuracy and industrial applicability of the CFD dataset.

## Usage Notes

### AI-Driven Use Cases

This dataset serves as a valuable resource for advancing research and development in computational fluid dynamics (CFD) and machine learning. Below are key use cases where this dataset can be leveraged:

- **Parameter Tuning**. Parameter optimization for linear solver configurations remains a critical challenge in modern CFD solvers. Algorithms like AMG (Algebraic MultiGrid) often expose over thirty tunable parameters that can significantly impact the solver’s convergence rate and computational efficiency^27– 29^. Our dataset contains multiple linear systems generated from a consistent vessel geometry across successive time steps, capturing the evolving nature of CFD simulations. Researchers can systematically test solver algorithms on these related systems to identify parameter combinations that optimize performance not just for individual matrices, but for entire families of linear systems arising from the same physical problem. This enables the development of robust, generalizable tuning strategies for real-world CFD workflows.
- **Neural Preconditioner Operator**. Preconditioners play a pivotal role in accelerating the iterative solution of large-scale linear systems by improving their spectral properties. While a well-chosen preconditioner can dramatically reduce convergence iterations, traditional construction methods (e.g., incomplete LU factorization^30,31^, AMG) often incur significant computational overhead that dominates the total solver time. Though our dataset does not include precomputed preconditioners, it provides a structured collection of linear systems derived from vessel-based CFD simulations. Researchers can leverage these systems to first generate high-performance preconditioners using established numerical methods, then train neural operators to learn mappings from system matrices (or problem metadata, such as time steps or mesh properties) to optimized preconditioner approximations^32,33^. Crucially, the dataset’s consistency across time steps and geometries enables learning preconditioning strategies that generalize across related physical configurations, rather than focusing on isolated linear systems.
- **PINNs**. PINNs hold significant potential for advancing computational simulation^15,21,34^. Unlike traditional methods, PINNs bypass the need to iteratively solve linear equation systems, instead of directly predicting flow fields or other target variables through neural networks. However, to ensure accuracy, training PINNs requires explicit integration of physical constraints derived from the problem’s governing equations, boundary conditions, and domain-specific parameters. Our dataset addresses this need by incorporating both the physical context of the simulations (e.g., governing equations, boundary conditions, and material properties) and numerical details such as the discretized matrix operators, right-hand side (RHS) vectors, and time-step-resolved solutions. By coupling raw numerical data (matrix/RHS/solution pairs) with their underlying physical descriptions, this framework enables robust training of PINNs, ensuring adherence to first principles while maintaining computational efficiency.

### Other Use Cases

Beyond the AI-driven applications, our dataset also supports traditional research. Management provides the organizational backbone for evaluation and visualization; evaluation results are often explored visually for deeper insight, while visualization can pinpoint subsets for further quantitative analysis.

- **Scientific Data Management**. Scientific data management is a specific research topic in the field of database and data management^35,36^. Compared with traditional databases, scientific databases^37– 39^ has been less focused by the field of database research, an important reason is the lack of the requirement of managing large scale, scientific data. By providing rich CFD data as a scientific dataset, one can use this dataset to effectively evaluate the performance of scientific data management.
- **Solver Performance Evaluation and High-Performance Computing**. Similar to established matrix collections^17,25^, our dataset supplies a diverse set of sparse matrices (**A**) and right-hand side vectors (***b***) from industrial CFD, varying in sparsity pattern, condition number, and problem size. This enables rigorous benchmarking of both direct solvers (e.g., LU decomposition^40^) and iterative methods (e.g., conjugate gradient^41^, GMRES^42^). Within the supercomputing community, optimizing such kernels has been extensively studied from algorithmic^43– 45^ and energy-efficiency^46– 49^ viewpoints. By providing matrices from realistic CFD applications, our dataset offers a faithful industrial benchmark for evaluating these academic methods.
- **Post-processing Analysis and Visualization**. High-fidelity visualization of physical fields-such as vorticity contours, streamline animations, and scalar field renderings-represents a distinct research area in computational science, requiring careful balancing of accuracy, interpretability, and computational efficiency^50–52^. Our dataset facilitates this endeavor by providing time-resolved data for five distinct physical parameters (e.g., velocity components, pressure, temperature) across multiple time steps, enabling reconstruction of derived physical fields (e.g., vorticity, turbulent kinetic energy). By including raw and post-processed quantities, the dataset serves as a benchmark for evaluating visualization algorithms, allowing quantitative assessment of their geometric fidelity (e.g., contour sharpness), temporal coherence (e.g., smoothness of animations), and perceptual effectiveness (e.g., clarity in feature identification).

## Data Availability

The dataset is publicly available at the SciDB repository (https://www.scidb.cn/en), under the digital object identifier 10.57760/sciencedb.23682.

## Code availability

The CFD software MarineFlow, used to generate the dataset, is available on the official CSSRC platform (https://www.taihulab.com.cn/info/209.html). Users with a background in CFD can generate their own datasets using this software. The scripts for decrypting, reformatting, and analyzing the data records are released as open-source projects on GitHub:

• We provide a Python program for decrypting the .csr matrix files into matrices **A** and RHS vectors ***b***, which are stored using standard *Matrix Market* format (with .mtx extension). (https://github.com/ft1573734/Matrix_Converter).

• We provide a Python program for decrypting the .dat post-processing files into the data structure described in Section *Methods*. (https://github.com/ft1573734/Dat_Data_Decoder).

• We provide a CUDA C/C++ program for efficiently estimating the matrix features enumerated in Section *Technical Validation*. (https://github.com/ft1573734/Matrix_Features).
